# The EBV-Encoded Oncoprotein, LMP1, Induces an Epithelial-to-Mesenchymal Transition (EMT) via Its CTAR1 Domain through Integrin-Mediated ERK-MAPK Signalling

**DOI:** 10.3390/cancers10050130

**Published:** 2018-05-01

**Authors:** Mhairi A. Morris, Louise Laverick, Wenbin Wei, Alexandra M. Davis, Samantha O’Neill, Liam Wood, Jack Wright, Christopher W. Dawson, Lawrence S. Young

**Affiliations:** 1 School of Sport, Exercise and Health Sciences, Loughborough University, Loughborough LE11 3TU, UK; M.A.Morris@lboro.ac.uk; 2 The Department of Medicine, Clinical Sciences Building, The Royal Melbourne Hospital, University of Melbourne, Parkville VIC 3010, Australia; louise.laverick@mh.org.au; 3 Sheffield Institute of Translational Neuroscience, University of Sheffield, 385a Glossop Road, Sheffield S10 2HQ, UK; w.wei@sheffield.ac.uk; 4 Institute of Cancer and Genomic Sciences, College of Medicine & Dentistry, University of Birmingham, Birmingham B15 2TT, UK; c.w.dawson@bham.ac.uk; 5 Faculty of Health and Life Sciences, De Montfort University, Leicester LE1 9BH, UK; alexandramdavis1@gmail.com (A.M.D.); samantha.oneill@hotmail.com (S.O.); liamwood94@live.com (L.W.); jackwright71@hotmail.co.uk (J.W.); 6 Warwick Medical School, Gibbet Hill Campus, University of Warwick, Coventry CV4 7AL, UK

**Keywords:** EBV, LMP1, NPC, EMT, ERK-MAPK, PI3-Kinase, Src family kinases, β1 integrins

## Abstract

The Epstein–Barr virus (EBV)-encoded latent membrane protein 1 (LMP1) oncogene can induce profound effects on epithelial growth and differentiation including many of the features of the epithelial-to-mesenchymal transition (EMT). To better characterise these effects, we used the well-defined Madin Darby Canine Kidney (MDCK) epithelial cell model and found that LMP1 expression in these cells induces EMT as defined by characteristic morphological changes accompanied by loss of E-cadherin, desmosomal cadherin and tight junction protein expression. The induction of the EMT phenotype required a functional CTAR1 domain of LMP1 and studies using pharmacological inhibitors revealed contributions from signalling pathways commonly induced by integrin–ligand interactions: extracellular signal-regulated kinases/mitogen-activated protein kinases (ERK-MAPK), PI3-Kinase and tyrosine kinases, but not transforming growth factor beta (TGFβ). More detailed analysis implicated the CTAR1-mediated induction of Slug and Twist in LMP1-induced EMT. A key role for β1 integrin signalling in LMP1-mediated ERK-MAPK and focal adhesion kianse (FAK) phosphorylation was observed, and β1 integrin activation was found to enhance LMP1-induced cell viability and survival. These findings support an important role for LMP1 in disease pathogenesis through transcriptional reprogramming that enhances tumour cell survival and leads to a more invasive, metastatic phenotype.

## 1. Introduction

Epstein–Barr virus (EBV) is a human herpesvirus that infects over 90% of the worldwide population. This ubiquitous virus encodes a number of latent gene products involved in establishing latent viral infection, long-term episomal maintenance, and cellular transformation in the human host. As such, infection with EBV is associated with a number of B cell and epithelial malignancies, many of which display distinct geographical distribution and exhibit other co-operative causal factors [1]. Nasopharyngeal carcinoma (NPC) is a relatively rare head and neck cancer on the global scale, but geographically, has particularly high rates of incidence in Southeast Asia due to confounding causal factors, including dietary, environmental and genetic risk factors [2].

Amongst the latent genes encoded by EBV, latent membrane protein 1 (LMP1) is considered the major oncoprotein on account of its ability to transform B cells and rat fibroblasts in vitro, rendering them tumourigenic in nude mice [3]. Despite the broad variability in reported levels of LMP1 expression in NPC biopsies [4], it is widely believed that expression of LMP1 at the early stages of disease pathogenesis may play an important role in malignant transformation and the recruitment of the inflammatory infiltrate characteristic of this lymphoepithelioma [5]. LMP1 behaves as a constitutively active tumour necrosis factor (TNF) receptor, signalling via numerous pathways commonly deregulated in cancer including NF-κB1 and NF-κB2, ERK-MAPK, PI3K/Akt, JNK/SAPK, p38-MAPK and activin A/TGFβ [6,7]. This deregulation in cell signalling by LMP1 results in profound morphological alterations in both epithelial cells, such as through the epithelial-to-mesenchymal transition (EMT) [8,9,10] and in B cells, such as the multinuclearity observed in Hodgkin Reed Sternberg cells from downregulation of shelterin protein components [11].

EMT is the transcriptional reprogramming of epithelial cells characterised by decreased adhesion and enhanced migration and invasion [12]. The EMT programme is usually reserved for gastrulation and neural crest cell delamination in embryonic development, but is inappropriately reactivated by cancer cells in tumourigenesis [13,14]. During EMT, epithelial cells transdifferentiate to acquire a mesenchymal phenotype, accompanied by the loss of cell–cell adherens junctions, loss of cell polarity, and actin cytoskeleton remodeling [12]. Simultaneously, cells undergoing EMT exhibit increased expression of mesenchymal markers and enhanced migratory abilities, mediated by alterations in cell-extracellular matrix (ECM) interactions [15]. The classic characteristic observed in cancer-related EMT is that of cadherin switching: expression of epithelial cadherin (E-cadherin) is lost, along with expression of tight junction, adherens junction and desmosomal proteins; whilst the neuronal cadherin (N-cadherin) is inappropriately re-expressed, along with other mesenchymal markers [16,17].

Numerous pathways have been implicated in the progression of EMT, including signalling via cell surface integrin receptors, the TGFβ signalling pathway, and the pleiotropic JNK/SAPK and MAPK signalling pathways [18]. The most effective and best understood of these is TGFβ; however, there is significant crosstalk between the pathways involved, mediated via transcription factors including Slug and Snail, Twist and ZEB1/2, which are induced by TGFβ-Smad signalling. In concert, these signal transduction pathways orchestrate the progression of an EMT programme [19,20]. In addition to canonical TGFβ signalling via the Smad family of transcription factors, non-canonical Smad-independent TGFβ signalling has also been implicated in EMT via crosstalk with other pathways, including the Ras-Raf-MEK-ERK kinase module and PI3K/Akt signalling [21]. This is particularly relevant to Madin Darby Canine Kidney (MDCK) cells, where TGFβ treatment has been shown to cooperate with oncogenic Ras to induce an EMT [22].

αvβ5 integrins bind to the arginine-glycine-aspartic acid (RGD) peptide motif in ECM proteins such as fibronectin, vitronectin and laminin [23], and subsequently transmit intracellular signalling via mediators such as integrin linked kinase (ILK) [10,24] and focal adhesion kinase (FAK) [25,26]. In addition to TGFβ-mediated activation of PI3K/Akt signalling, Akt can also be phosphorylated by integrin-mediated ILK activation [27].

Additional layers of complexity surrounding EMT are evident in the crosstalk between TGFβ and ERK-MAPK signalling, and furthermore the crosstalk between ERK-MAPK and JNK/SAPK signalling. Such intricacies are likely cell-type and context-dependent. In brief, studies in normal mouse mammary epithelial cells revealed a role for ERK-MAPK in TGFβ-mediated EMT [28]. Moreover, constitutive JNK/SAPK activation promoted EMT in human mammary epithelial cells, an effect that required ERK-MAPK activity [29]. Clearly, there are multiple mechanisms by which a population of tumour cells can undergo EMT, and thus a viral oncoprotein that engages many of these same signalling pathways may well utilise more than one to elicit its tumourigenic effects.

Previous studies have demonstrated the propensity of LMP1 to transform MDCK epithelial cells by promoting an EMT [30], a phenomenon attributable to the transcriptional repressor, Twist, in this cell line [8]. Further studies have corroborated LMP1’s ability to induce EMT in other cell lines, including breast epithelia [31], lung epithelia [32] and nasopharyngeal epithelia [30,33,34]. In NPC biopsies, LMP1 expression correlates with overexpression of the transcriptional repressor, Snail [9]. In the same study, expression of LMP1 in a human nasopharyngeal epithelial cell line-induced EMT in a Snail-dependent mechanism. More recently, LMP1 was found to promote EMT and activate cadherin switching from E-cadherin to K-cadherin (cadherin 6) in NPC [35].

In the current study, MDCK cells expressing a panel of wildtype and LMP1 mutants defective for the two primary signalling domains of LMP1 were used to identify the C-terminal activatory region 1 (CTAR1) as the signalling domain responsible for LMP1-mediated EMT induction. Pharmacological inhibition of ERK-MAPK, PI3K/Akt and Src family kinases (SFKs) elicited a reversal of the EMT phenotype in wildtype LMP1-expressing cells; however, inhibition of canonical Smad-dependent TGFβ signalling had no impact on LMP1-medated EMT morphology in MDCK cells. Subsequent cDNA microarray analysis was used to generate a global gene expression profile for each cell line, identifying groups of EMT-related genes deregulated by LMP1, including those pertaining to the integrin and MAPK signalling pathways. Interestingly, LMP1-mediated ERK-MAPK and FAK phosphorylation appears to require β1 integrin–fibronectin ligand interactions. Further downstream investigations revealed a role for LMP1-mediated β1 integrin activation in protecting epithelial cells from suspension-induced apoptosis (“anoikis”), since blocking β1 integrin signalling reduced cell viability in LMP1-expressing cells held in suspension. Taken together, these findings support the role for LMP1 in the early stages of NPC pathogenesis, by driving an EMT-like programme of transcriptional reprogramming through engaging a network of overlapping signalling pathways in a complex fashion, to promote survival and metastasis.

## 2. Results

### 2.1. LMP1 Induces EMT in MDCK Cells via CTAR1

When expressed in human epithelial cells, LMP1 induces profound morphological changes, inducing a breakdown in cell–cell contact and the loss of epithelial cell characteristics [36]. These effects are particularly evident in the canine kidney epithelial cell line, MDCK, where LMP1 induces morphological alterations reminiscent of cells undergoing an epithelial-to-mesenchymal transition (EMT) [8,10]. MDCK cells are a well-established model for studying the signalling processes involved in the regulation of EMT and we previously engineered these cells to stably express full-length LMP1 or mutants carrying defective CTAR1, CTAR2 or CTAR1/2 signalling domains (denoted hereafter as wildtype, CTAR1+/2−, CTAR1−/2+ and CTAR1−/2−, respectively) [37].

**Figure 1:**
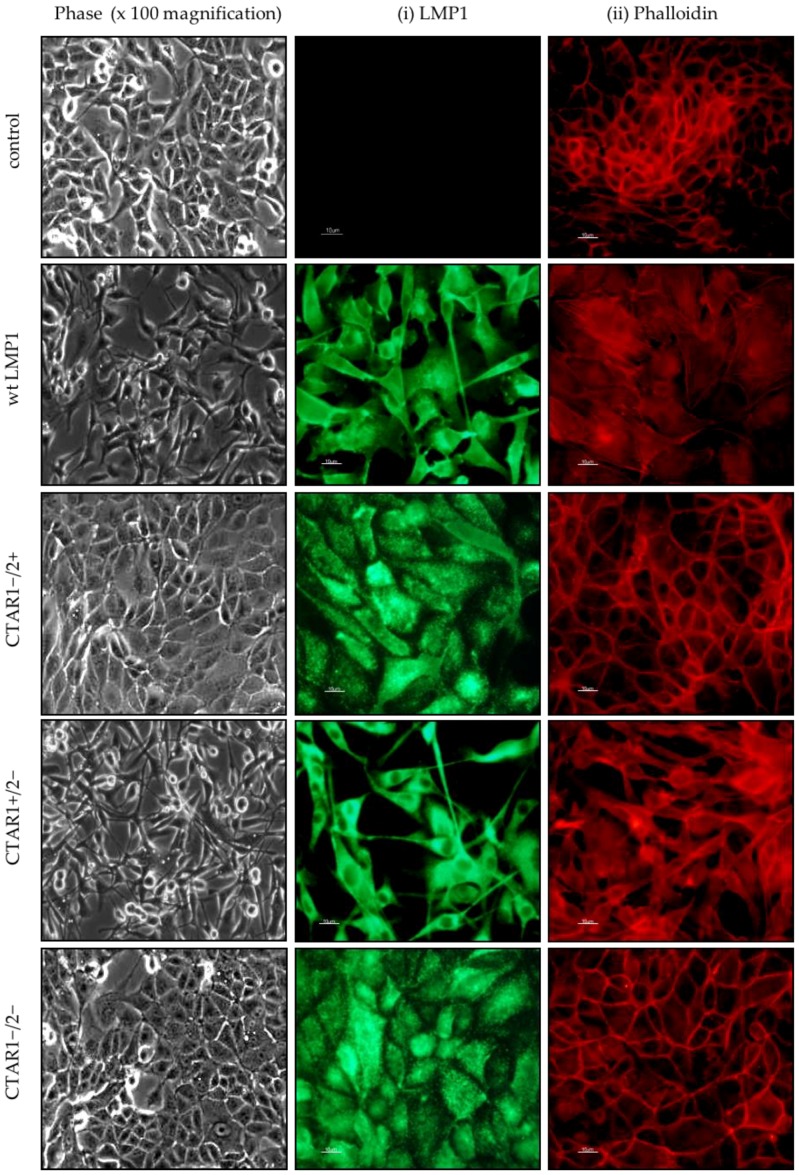
Stable LMP1 expression alters cell morphology. Phase contrast images of Madin Darby Canine Kidney (MDCK) epithelial cells stably expressing the neomycin control, wildtype LMP1 (wt LMP1), CTAR1−/2+ LMP1, CTAR1+/2− LMP1 and CTAR1−/2− LMP1 mutants were captured on a Zeiss digital microscope at 100× magnification. (i) Immunofluorescence staining using an LMP1-specific monoclonal antibody, CS2, confirmed ubiquitous expression of LMP1 in the wildtype and CTAR1/2 domain mutants; (ii) Actin stress fibre formation was confirmed in the wildtype and CTAR1+/2− mutant LMP1-expressing cells only, as visualised using TRITC-conjugated Phalloidin Ig. Bar = 10 μm.

Consistent with earlier observations, MDCK cells expressing wildtype LMP1 underwent an EMT after two weeks of continual subculturing, after which time cells took on a fusiform appearance and “fibroblastic” morphology with distinct alterations in actin stress-fibre formation (compare wildtype LMP1 (pre-EMT) with wildtype LMP1 (post-EMT) in Figure 1). Whilst cells lacking the CTAR1 domain of LMP1 (both the CTAR1−/2+ mutant and the CTAR1−/2− double mutant) were indistinguishable from control cells, cells expressing an intact CTAR1 domain (CTAR1+/2− LMP1 mutant) were similar in appearance to wildtype LMP1-expressing cells, suggesting that the CTAR1 domain of LMP1 is both necessary and sufficient to induce an EMT in MDCK cells.

### 2.2. Critical Roles for ERK-MAPK, PI3-Kinase, Src Family Kinases (SFKs) But Not TGFβ Signalling Pathways in LMP1-Mediated EMT

During the EMT process, cells adopt a number of mesenchymal features including enhanced cell invasion and migration [38]. Previous studies have highlighted a role for LMP1 in modulating cell migration in an ERK-dependent manner, which is activated by the CTAR1 domain [37,39].

**Figure 2:**
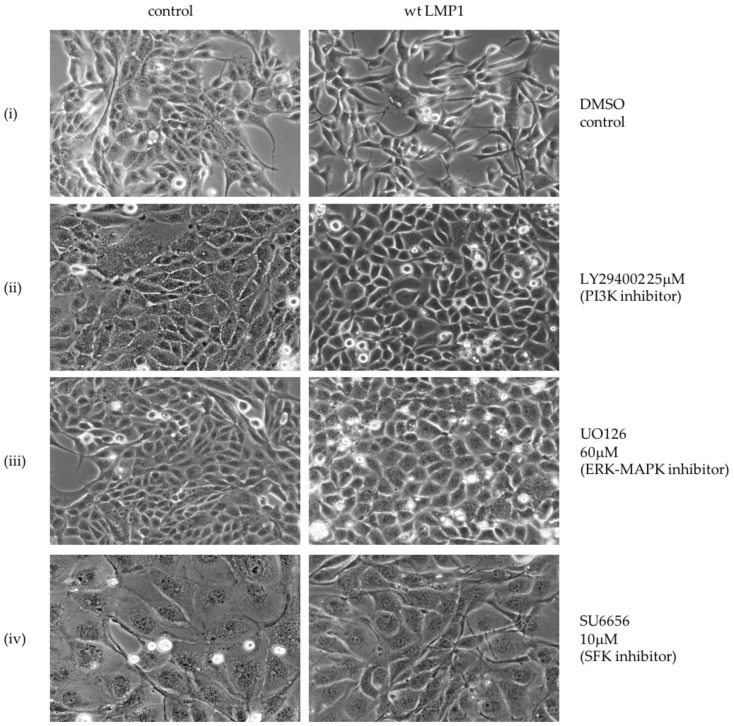

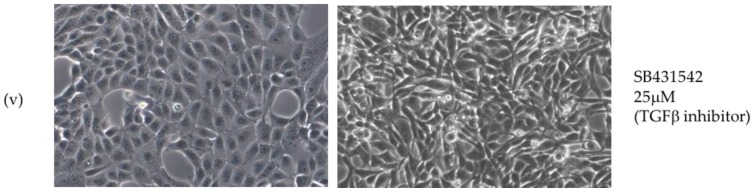
Specific chemical inhibitors “reverse” the transformed morphology of LMP1-expressing cells. Serum-starved MDCK control and wildtype LMP1 stably expressing cells were treated for 24 h with (**i**) the inert carrier solvent, dimethyl sulphoxide (DMSO), to a final concentration of 1:1000; or pharmacological inhibitors to (**ii**) PI3K (LY294002) to a final concentration of 25 μM; (**iii**) ERK-MAPK (UO126) to a final concentration of 60 μM; (**iv**) Src family kinases (SFKs; SU6656) to a final concentration of 10 μM; or (**v**) canonical Smad-dependent TGFβ (SB431542) to a final concentration of 25 μM. Representative phase contrast images were captured on a Zeiss digital microscope at 100× magnification.

LMP1 itself engages a number of the cell signalling pathways capable of inducing an EMT, including the ERK-MAPK, PI3K/Akt and TGFβ signalling pathways, and has also been shown to modulate cell adhesion via β1 integrin signalling [7,37,40]. Using selective pharmacological inhibitors of key pathway effectors to investigate the contribution of each of these pathways in turn, their effects on the morphology of MDCK cells stably expressing LMP1 revealed a reversal of the EMT phenotype by the MEK inhibitor (UO126), the PI3K inhibitor (LY294002) and the SFK inhibitor (SU6656; Figure 2). However, despite well-established data supporting the role of TGFβ in the EMT process [21], inhibition of TGFβ signalling using the type I receptor inhibitor (SB431542) failed to have any impact on the morphology of the LMP1-expressing cells, suggesting that TGFβ signalling is not implicated in the LMP1-mediated EMT phenotype in MDCK cells. Regardless, it is evident that LMP1 is mediating the EMT phenotype in MDCK cells via a kinase-dependent mechanism involving ERK-MAPK, PI3K and SFKs—pathways that are commonly induced in response to integrin–ligand interactions.

### 2.3. Global Gene Expression Analysis Reveals a Distinct Role for CTAR1 in Deregulating Genes from Key Signalling Pathways Implicated in EMT

With the release of the canine genome sequence, the dog is now amenable to comparative genomic analysis. Initial assessments of the canine genome suggest that the canine and human lineages are more similar than human and rodent lineage in terms of both nucleotide divergence and rearrangements [41].

**Figure 3:**
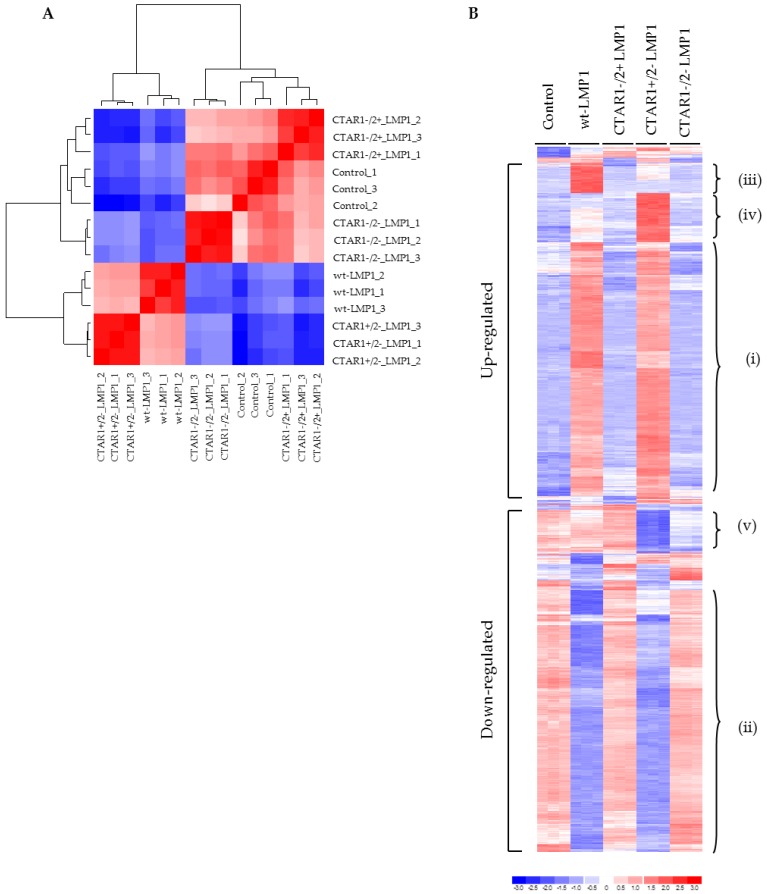
Heatmaps of Pearson correlation and relative expression of probe sets differentially expressed among the five groups identified by multiclass SAM analysis. (**A**) Heatmap of Pearson correlation (ranging from 0.948 to 1) of the 15 individual arrays utilised in this study. The relative expression of all probe sets was exploited in the calculation of Pearson correlation; (**B**) The top 1021 probe sets differentially expressed among the five groups were identified by multiclass significance analysis of microarrays (SAM) analysis with the criteria of fold change greater than 2% and 0% false discovery rate (FDR). Gene expression heatmaps were generated using dChip (http://www.dchip.org) with the default settings. The heatmap highlights differences in the induction of various genes, which is noted by the difference in the intensity of the bars: blue (low) or red (high). A high-level examination of the heatmap revealed genes whose expression could be clustered into those that were: (i) significantly upregulated in wildtype LMP1 and CTAR1+/2− LMP1-expressing cells; (ii) significantly downregulated in wildtype LMP1 and CTAR1+/2− LMP1-expressing cells; (iii) significantly upregulated in wildtype LMP1-expressing cells only; (iv) significantly upregulated in CTAR1+/2− LMP1-expressing cells only; (v) significantly downregulated in CTAR1+/2− LMP1-expressing cells only.

Following on from the observations detailed in Figure 1, microarray technology was utilized to analyse the impact of LMP1 on EMT-related gene transcription and evaluate the contribution of the two C-terminal activatory regions of LMP1 on the transcriptional profile in MDCK cells. A sample correlation heatmap (Figure 3A) was constructed to show Pearson correlation calculated using all probe sets between 15 individual samples employed in this study. It shows that the biological triplicates in each group correlate strongly with each other, and that the five groups form two clusters: one containing wildtype LMP1 and CTAR1+/2− mutant LMP1-expressing cells, the other containing the remaining three groups. Therefore, cells expressing an intact CTAR1 domain (CTAR1+/2− LMP1 mutant) are similar to wildtype LMP1-expressing cells not only in appearance, but also in gene expression profile.

Multiclass significance analysis of microarrays (SAM) analysis [42] was used to identify the top 1021 probe sets differentially expressed among the five groups with the criteria of fold change greater than 2% and 0% false discovery rate (FDR). Figure 3B shows the gene expression heatmap generated using dChip (http://www.dchip.org) with the default settings, and demonstrates key differences in global gene expression patterns between control cells and the LMP1-expressing panel of cell lines. For example, areas within the heatmap identify genes whose expression is: (a) significantly upregulated in wildtype LMP1 and CTAR1+/2− mutant LMP1-expressing cells (Figure 3Bi); (b) significantly downregulated in wildtype LMP1 and CTAR1+/2− mutant LMP1-expressing cells (Figure 3Bii); (c) significantly upregulated in wildtype LMP1-expressing cells only (Figure 3Biii); (d) significantly upregulated in CTAR1+/2− mutant LMP1-expressing cells only (Figure 3Biv); or (e) significantly downregulated in CTAR1+/2− mutant LMP1-expressing cells only (Figure 3Bv).

Probe sets differentially expressed between each of the four groups of cells expressing wildtype LMP1, CTAR1+/2−, CTAR1−/2+ and the CTAR1−/2− double-mutant derivatives and the control were identified using the rank products method [43,44] with the criteria of percentage of false-positives less than 10% and absolute fold change ≥1.5. Full lists of selected differentially upregulated and downregulated genes with their Entrez Gene ID can be viewed in Supplementary Tables S1 and S2. The minus (−) sign indicates downregulation, whereas “NS” indicates no significant change in expression.

Having established that substantial blocks of genes were differentially regulated by wildtype LMP1 and CTAR1+/2− mutant LMP1-expressing cell lines, a second heatmap was generated using certain genes identified in Supplementary Figure S1. Here, genes were selected for their proven or potential involvement in the induction of an EMT phenotype. In total, 39 downregulated genes and 59 upregulated genes were selected. Genes were listed according to their main function. Category analysis showed that LMP1 expression appeared to influence multiple cellular processes including EMT, wound response, apoptosis, inflammation and angiogenesis, signal transduction and cell adhesion, amongst others.

A number of genes were identified as being involved in the formation of adherens junctions, including E-cadherin, Slug, RhoA and focal adhesions, including many of the alpha and beta integrin family subunits (α2, α5, αv, β1, β4, β6). Other differentially regulated genes involved in focal adhesions include those encoding many ECM proteins, such as fibronectin, collagens (significantly COL1A2) and laminins (significantly LAMC2). Alterations in the balance of expression of many of these ECM components is associated with a shift from epithelial-to-mesenchymal cell morphology. An upregulation in collagens and fibronectin results in a more motile and less adhesive phenotype, an effect that is also integrin-dependent. Supplementary Figure S2 shows the heatmaps generated for those genes implicated in adherens junctions and focal adhesions, with further details tabulated in Supplementary Table S3.

Table 1 summarises the key gene changes mapped to the CTAR1 domain of LMP1 in relation to EMT, MAPK and integrin signalling, many of which were selected for further validation.

### 2.4. Stable Expression of LMP1 in MDCK Cells Is Accompanied by the Complete Loss of Epithelial Markers and Upregulation of Mesenchymal Markers

EMT is an extreme form of cellular plasticity defined by the loss of epithelial morphology, dissolution of cell–cell contacts, actin filament remodeling and the acquisition of a mesenchymal morphology. In vitro observations classically include the downregulation of epithelial markers, such as E-cadherin, and the concomitant upregulation of mesenchymal markers, such as N-cadherin. This phenomenon, known as cadherin switching, is one mechanism by which a population of cells can separate from their neighbours, allowing them to invade and metastasise to a secondary site [16].

**Figure 4:**
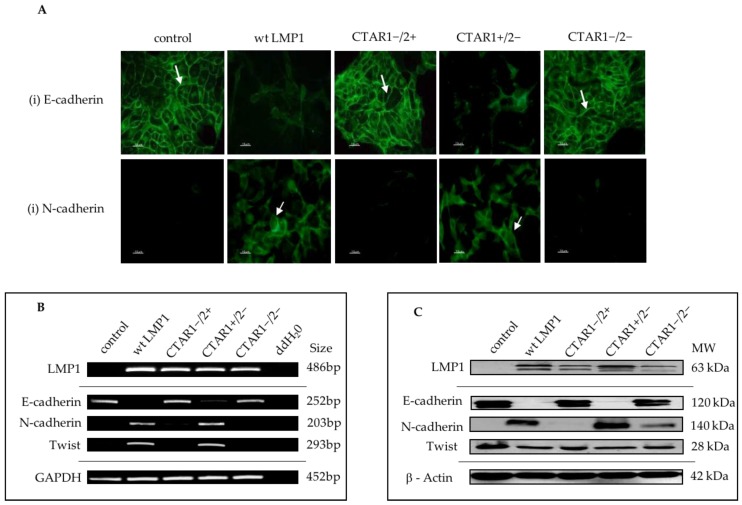
Characterisation of expression of EMT markers in LMP1-expressing MDCK cells. (**A**) Immunofluorescence staining confirmed downregulation of the epithelial marker, E-cadherin, as indicated by the white arrows, and a concomitant upregulation of the mesenchymal marker, N-cadherin, in wildtype LMP1 and CTAR1+/2− LMP1-expressing cells. Bar = 10 μm; (**B**) Reverse transcriptase polymerase chain reaction (RT-PCR) confirmed the loss of E-cadherin gene expression and the accompanied induction of N-cadherin gene expression in wildtype and CTAR1+/2− LMP1-expressing cells. The transcriptional repressor, Twist, was also upregulated in a CTAR1-dependent manner. The housekeeping gene for GAPDH was included as an internal loading control; (**C**) Western blotting further validated the changes in gene expression of both E-cadherin and N-cadherin at the protein level, but induction of Twist at the RNA level does not appear to be translated at the protein level. The structural protein, β-actin, was included as an internal loading control. N.B. All samples were collected as part of the same biological triplicate and therefore the same loading control and LMP1 expression control are used throughout.

In line with the morphological observations outlined in Figure 1, and the microarray data summarized in Figure 3, a selection of EMT-related genes were selected for further downstream validation. Using combinations of immunofluorescence staining (Figure 4A), reverse transcriptase polymerase chain reaction (RT-PCR; Figure 4B) and Western blotting (Figure 4C), the complete loss of E-cadherin expression was observed, along with the concomitant upregulation of the mesenchymal marker, N-cadherin, and the induction of the transcription factor, Twist, which is known to be implicated in LMP1-mediated EMT [8]. Further validation of numerous EMT markers are summarized in Supplementary Figures S3 and S4, including the downregulation of desomosomal cadherins, desmocolin (DSC2 and 3), desmoglein (DSG2 and 3), and desmoplakin (DPO), and tight junction proteins (ZO-1, Occludin) in LMP1-expressing cells compared to their control counterparts. In addition to the loss of epithelial markers, cells expressing wildtype LMP1 and the CTAR1+/2− LMP1 mutant also displayed a concomitant upregulation of mesenchymal markers associated with EMT, including vimentin and fibronectin, findings that support the notion that LMP1-mediated EMT is associated with “cadherin switching”.

Supplementary Figure S4C demonstrates the significantly lower levels of E-cadherin promoter activity in MDCK cells expressing wildtype LMP1 and the CTAR1+/2− LMP1 mutant, whereas activity was similar to control cells where there was no functional CTAR1 domain. Similarly, the upregulated expression of the mesenchymal marker, fibronectin, was also found to be a result of transcriptional deregulation in wildtype LMP1 and CTAR1+/2− mutant LMP1-expressing cells, since fibronectin promoter activity was enhanced in both wildtype and CTAR1+/2− mutant LMP1-expressing cells, a phenomenon that was not observed in the absence of a functional CTAR1 domain (Supplementary Figure S4D).

In many epithelial systems, the loss of E-cadherin and desmosomal expression occurs through transcriptional targeting of the E-cadherin promoter by the Snail family of transcription factors. Analysis of the microarray data revealed increased expression of a number of EMT-inducing transcription factors, notably Snail, Slug, zinc finger E-box binding homeobox 1 (ZEB1/TCF8, ZEB2/SIP), and Twist in MDCK cells expressing wildtype LMP1 or CTAR1+/2− mutant LMP1 compared to control cells or cells expressing the CTAR1−/2+ mutant LMP1 or the CTAR1−/2− double-mutant LMP1 (Figure 3A, Table 1). Further validation of the array predictions was performed using RT-PCR and Western blotting analysis, confirming increased levels of expression of SNAIL, SNAI2 (Slug), ZEB1, ZEB2 (SIP1) and Twist in MDCK cells expressing wildtype LMP1 and the CTAR1+/2− mutant LMP1 at the RNA level (Supplementary Figure S4A) and for Snail, Slug and Twist at the protein level (Supplementary Figure S4B).

### 2.5. LMP1 Deregulates Various Components of MAPK Signalling Implicated in EMT

A number of components of MAPK signalling pathways are important in driving EMT, and there is a significant amount of crosstalk between these (and other) signalling pathways. For example, both TGFβ and JNK/SAPK-mediated EMT have been shown to require ERK-MAPK signalling [28,29].

LMP1 activates extracellular signal-related kinases (ERK1/2), c-Jun NH_2_-terminal kinases (JNK1/2) and p38 MAPK, as well as PI3K/Akt [6], pathways which have all been implicated in the EMT response. The ERK kinases can be activated by diverse mechanisms, including ligation of receptor tyrosine kinases (RTKs), such as EGF, and cell adhesion molecules, including integrins. In general, ligand binding of these receptors leads to guanosine triphosphate (GTP) loading and activation of the small GTP-binding protein, Ras. Upon stimulation, activated Ras binds the MAPK kinase kinase (MAPKKK), Raf, which in turn phosphorylates and activates the MAPK kinases, MEK1/2 (MKK1/2) and ERK (ERK1/2). This cascade of kinases, also called the Ras-Raf-MEK-ERK kinase module, is often deregulated in cancer.

In response to growth factors, PI3K can be activated by receptor protein tyrosine kinases (RTKs) and non-RTKs. RTKs interact with the p85 regulatory subunit of PI3K, whilst the Ras protein directly interacts with the p110 catalytic subunit of PI3K in a GTP-dependent manner. PI3K activation in turn activates the serine/threonine kinase, Akt, that is involved in cell cycle progression, cell proliferation and prevention of apoptosis, and also plays a central role as an effector of EMT. Signalling via the PI3K/Akt pathway can lead to activation of the Rho-GTPases and also cooperates with TGFβ signalling during EMT [45].

**Figure 5:**
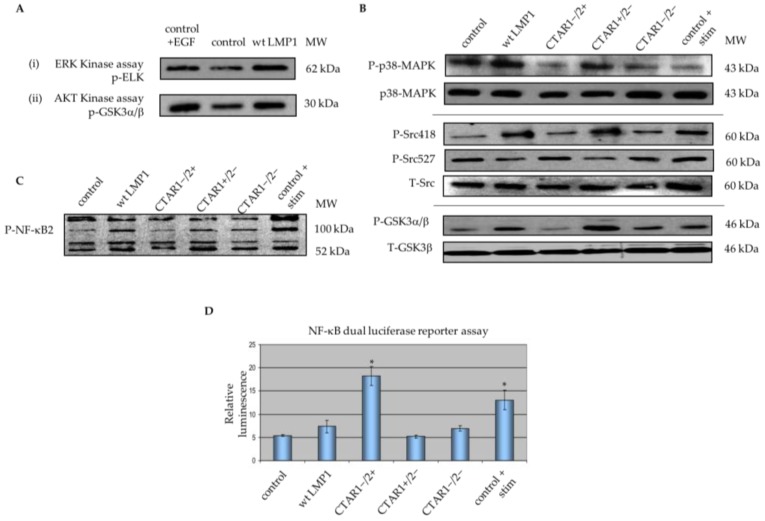
LMP1 deregulates various components of MAPK signalling implicated in EMT. (**A**) Non-radioactive kinase assays confirmed that LMP1 upregulates ERK/MAPK and Akt kinase activity by immunoprecipitation. Briefly, whole cell lysates from serum-starved cells were immobilised with: (i) threonine 202/tyrosine 204-phosphorylated p44/42 MAPK monoclonal antibody and reciprocally immunoblotted for serine 383-phosphorylated Elk-1; or (ii) Akt monoclonal antibody and reciprocally immunoblotted for serine 21/9-phosphorylated GSK3α/β; (**B**) Western blotting with phospho-specific antibodies for threonine 180/tyrosine 182-phosphorylated p38-MAPK, tyrosine 418/tyrosine 527-phosphorylated Src, and serine 21/9-phosphorylated glycogen-synthase kinase 3 α/β (GSK3α/β) confirmed activation of p38-MAPK and Src tyrosine kinase in wildtype and CTAR1+/2− LMP1-expressing cells; (**C**) Whole cell lysates from serum-starved cells were separated by SDS-PAGE and immunoblotted using a phospho-specific antibody against the p100 and p52 subunits of NF-κB2 for evidence of processed and non-processed forms of NF-κB2 (p100/p52). Control cells were stimulated with 100 ng/mL recombinant human tumour necrosis factor-α (TNFα) cytokine for 90 min to use as a control for NF-κB2 activation; (**D**) NF-κB activity in the panel of MDCK cells was examined by luciferase reporter assay. Control cells were stimulated using 100 ng/mL recombinant TNFα for 90 min prior to the assay. The histogram depicts the fold increase (mean ± SD; *n* = 3) in the levels of LMP1-mediated NF-κB luciferase reporter activity relative to that of corresponding cells transfected instead with a control vector (pGL2 basic), which are given an arbitrary value of 1. Asterisks indicate results significantly different from the LMP1-negative control (*p* < 0.01).

Previous studies have mapped ERK-MAPK and PI3K/Akt signalling to the CTAR1 domain of LMP1, as demonstrated by Western blotting using phospho-specific antibodies to components of each of these pathways [37]. A non-radioactive kinase assay confirmed that the observed increase in p44/42 MAPK (ERK1/2) phosphorylation correlated with increased p44/42 MAPK activity, and the increase in phosphorylated Akt protein also resulted in increased Akt kinase activity (Figure 5A). Furthermore, Western blotting demonstrates that the CTAR1 domain of LMP1 is also implicated in activating p38-MAPK signalling, as well as Src tyrosine kinase and GSK3β activity, both of which are downstream of PI3K/Akt, and ultimately, cell surface integrin receptors (Figure 5B). Further support is lent to these observations from the findings by Schramek and colleagues that EMT is reversed in the absence of constitutive MEK1 activity in renal epithelial MDCK-C7 cells, suggesting that the MEK1/2-ERK1/2 signalling module is a dominant signalling pathway involved in EMT in this cell type [46].

Microarray data revealed that LMP1 also deregulates a number of genes involved in MAPK signalling that may be implicated in the LMP1-mediated EMT phenotype, including NF-κB2, RASA1, IL-α, MAPK1/3 and MAP4K3 (Supplementary Figure S1 and Supplementary Table S1). LMP1 has previously been shown to induce expression of both the canonical (NF-κB1/p105) and non-canonical (NF-κB2/p100) signalling pathways [47]. Western blotting using an antibody specific to phosphorylated components of NF-κB2, p100 and p52, confirmed a role for CTAR1 in NF-κB2 activation (Figure 5C). The expression of both p100 and p52 protein subunits in control cells was greatly enhanced upon TNFα stimulation, with wildtype LMP1 and CTAR1+/2− mutant LMP1-expressing cells showing a modest induction of both the precursor (p100) and processed (p52) subunits relative to unstimulated control and LMP1-expressing cells lacking a functional CTAR1 domain. Additionally, use of the NF-κB luciferase reporter construct (3Enh-κB-ConALuc) confirmed the ability of LMP1 to enhance canonical NF-κB1 promoter activity (Figure 5D). All LMP1-expressing cells demonstrated a relative fold increase of NF-κB promoter activity when compared with the control cells. The CTAR2 domain of LMP1 shows significant augmentation of NF-κB signalling, with CTAR1−/2+ mutant LMP1-expressing cells demonstrating a 3.5-fold induction of NF-κB promoter activity relative to control and CTAR1+/2− mutant LMP1-expressing cells. This is also of a greater order of magnitude than that observed following TNFα stimulation of control cells (2.5-fold induction relative to unstimulated control cells) or in wildtype LMP1-expressing cells (1.5-fold induction relative to unstimulated control cells). Taken together, these results support the observation that the CTAR1 domain of LMP1 is important for activation of numerous components of the MAPK signalling pathways often deregulated in EMT, although further work is required to determine whether these pathways are indeed implicated in LMP1-mediated EMT.

### 2.6. LMP1 Deregulates the Expression of Multiple Genes in the Integrin Signalling Pathway Implicated in the Generation of an EMT

In addition to being stimulated by TGFβ signalling, various integrins (including αvβ3, αvβ5, αvβ6 and several β1 integrins) are able to bind latent TGFβ embedded within the ECM in the tumour microenvironment, thus activating TGFβ signalling and subsequently Src/FAK complex formation. The resultant loss in E-cadherin-dependent cell–cell adhesion promotes EMT [15]. Another class of proteins involved in integrin-mediated EMT are the urokinase (uPA)-type plasminogen activator receptor (uPAR) and its ligand, uPA. uPAR is a GPI-anchored receptor that is involved in regulating cell adhesion, migration and proliferation, and is known to contribute to EMT independently of the enzymatic activity of uPA [48]. uPAR can interact with β1, β2 and β3 integrins to regulate their activities. It also serves as an adhesion molecule, binding to the ECM protein vitronectin, and in so doing, can induce EMT [49]. Integrin-linked kinase (ILK) is a signalling component that is directly recruited to the cytoplasmic domains of β1 and β3 integrin subunits [50], and its activity is central to the processes of actin reorganisation, cell polarisation, spreading and migration [51].

Previous studies have shown that LMP1 expression correlates with fibronectin expression in nasopharyngeal carcinoma [52], and that functionally, LMP1-mediated fibronectin deposition facilitates epithelial cell adhesion and migration in an activin A/TGFβ and β1 integrin-dependent manner [7]. Similar observations in MDCK cells expressing wildtype LMP1 and the CTAR1+/2− LMP1 mutant confirm that the ability of LMP1 to induce fibronectin expression and promoter activity can be mapped to the CTAR1 domain (Figure 3B; and Figure S4D). Fibronectin is the major ligand for β1 and αv-containing integrin receptors [53,54]. Microarray data revealed significant upregulation of genes encoding fibronectin, α5 and β1 integrins in both wildtype LMP1 and the CTAR1+/2− LMP1 mutant, and a significant downregulation of genes encoding α6 and β4 integrins (Supplementary Figure S5A and Table 1), along with elevated levels of ILK protein expression (Supplementary Figure S5B).

**Figure 6:**
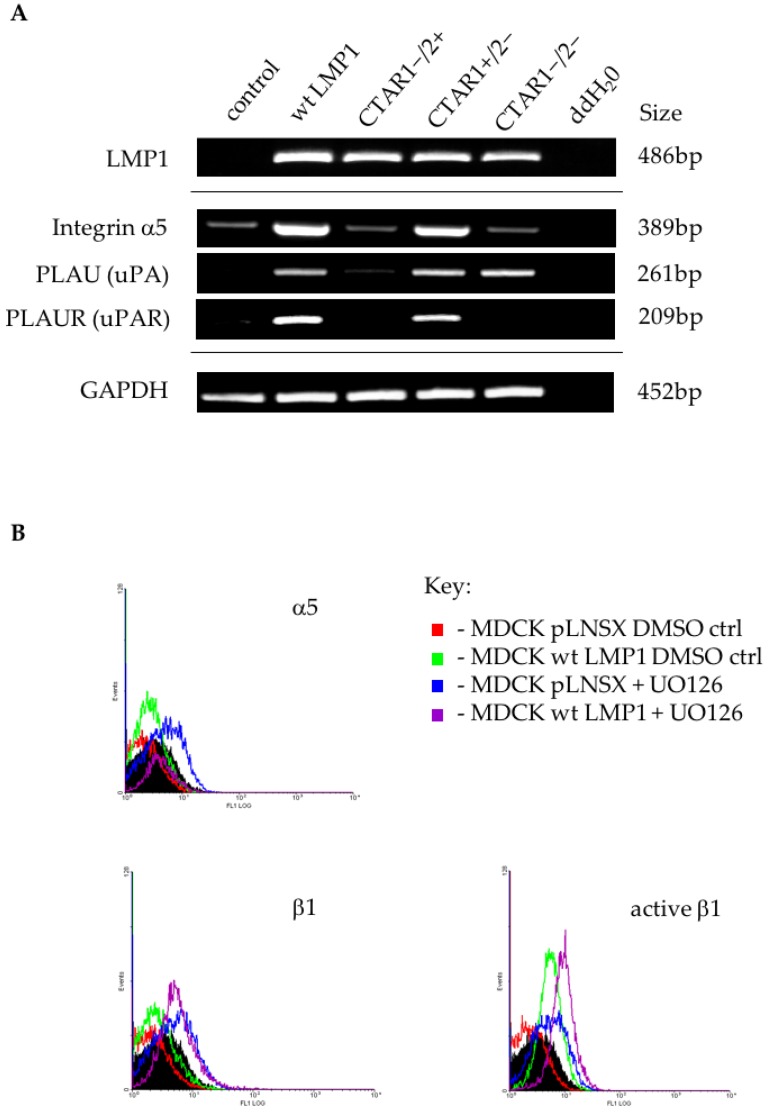
LMP1 deregulates integrin subunit expression. (**A**) RT-PCR confirmed the upregulation of α5 integrin subunit, as well as urokinase plasminogen activator (uPA) and its receptor, uPAR in wildtype and CTAR1+/2− LMP1-expressing cells; (**B**) Flow cytometric analysis confirmed an increase in basal levels of α5 integrin expression on the cell surface of LMP1-expressing cells, but very little increase in basal levels of β1 integrin. Using an antibody that specifically recognises β1 integrin in its active conformation demonstrated an increase in β1 integrin activation on the cell surface of LMP1-expressing cells. This was unaffected by inhibition of ERK-MAPK signalling, since addition of 60 μM UO126 had no significant effect on cell surface expression of these integrin subunits, active or otherwise.

Further microarray validation was performed using RT-PCR analysis on RNA extracted from the panel of MDCK cells, confirming increased expression of the α5 integrin subunit (Figure 6A); however, flow cytometric analysis did now show significantly elevated levels of α5 integrin expression on the surface of wildtype LMP1-expressing cells (Figure 6B). Although the array data did now show altered β1 integrin expression in response to LMP1, and flow cytometry confirmed that there was no significant difference in the basal levels of β1 integrin expression on the surface of wildtype LMP1-expressing cells (Figure 6B), using an antibody that specifically recognises active β1 integrins demonstrated elevated levels of active β1 on the surface of MDCK cells expressing wildtype LMP1, suggesting that integrin activity is regulated by LMP1 in a more complex manner than mere transcriptional regulation (Figure 6B). In addition to LMP1 deregulating the expression and/or activation state of these integrin subunits, RT-PCR also demonstrated the ability of LMP1 to induce both uPA and uPAR expression via its CTAR1 domain (Figure 6A). Since uPAR is known to interact with β1 integrins, this may reflect an additional mechanism by which LMP1 is able to modulate cell adhesion and migration, and drive the EMT process; however, further studies are required before drawing any conclusions in this vein.

The microarray data also revealed that αv integrin was upregulated in the wildtype LMP1-expressing cells only, but downregulated in the CTAR1−/2+ LMP1 mutant, and that α6 and β4 integrin subunits were downregulated in LMP1-expressing cells (Supplementary Figure S1). RT-PCR confirmed the decrease in α6 and β4 integrin gene expression in wildtype LMP1-expressing cells. This increase in αv expression in the wildtype LMP1-expressing cells was also validated at the RNA level, but contrary to the array data, RT-PCR also revealed a slight increase in the CTAR1+/2− LMP1 mutant (Supplementary Figure S5A). Similarly, the array data demonstrated no change in expression levels for the β3 subunit in the wildtype LMP1-expressing cells, which was validated by RT-PCR; however, the CTAR1+/2− LMP1 mutant displayed an increase in β3 integrin expression by RT-PCR, despite being unchanged on the array data (compare Supplementary Table S3 with Supplementary Figure S5A). These slight anomalies notwithstanding, which are likely due to the dynamic nature of gene expression in a population of cells at any given moment in time, the data presented herein support the ‘bigger picture’ that LMP1 deregulates a number of genes and signalling pathways implicated in generating an EMT via its CTAR1 domain.

### 2.7. Ligand-Induced β1 Integrin Signalling Facilitates LMP1-Mediated ERK and FAK Phosphorylation, and Protects Epithelial Cells from Anoikis

β1 integrins can promote cell survival through coordinating the signals transduced via FAK, Src, ERK-MAPK and PI3K/Akt pathways [55]. FAK is activated by direct interaction with the cytoplasmic domain of β1 integrins at the sites of focal adhesion, which mediates survival through phosphorylation-dependent activation of several downstream molecules, including ERK-MAPK [56] and SFKs [57,58]. Since LMP1 is able to activate β1 integrins on the surface of epithelial cells [7] and can modulate cell motility via ERK-MAPK in MDCK cells [37], the interplay between the two pathways was investigated further.

**Figure 7:**
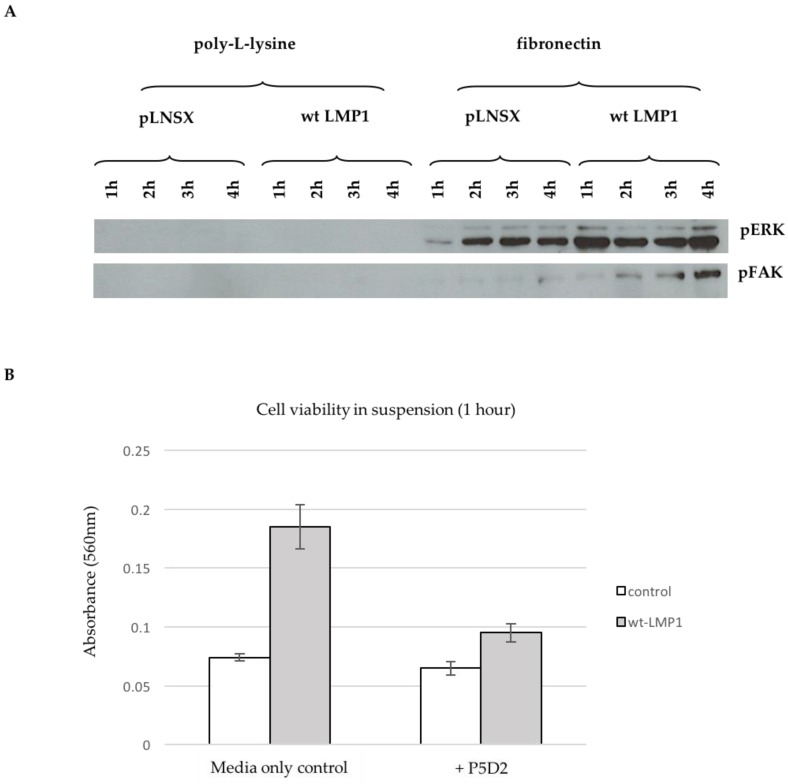
Ligand-induced β1 integrin signalling facilitates LMP1-mediated ERK and FAK phosphorylation, and protects epithelial cells from anoikis. (**A**) Cells were held in single-cell suspension for one hour and then plated onto poly-L-lysine or fibronectin-coated plates for one, two, three and four hours, respectively, prior to harvesting protein lysates. Western blotting with antibodies that specifically recognise threonine 202/tyrosine 204-phosphorylated p44/42 MAPK and tyrosine 397-phosphorylated FAK revealed the requirement for β1 integrin ligation for LMP1-mediated ERK-MAPK and FAK activity; (**B**) Control and wildtype LMP1-expressing cells were held in single-cell suspension for one hour by plating onto polyHEMA-coated plates prior to assessing cell viability by MTT assay, absorbance read at 560 nm. The histogram displays the average of technical triplicates for each sample, and is a representative of biological triplicate experiments.

Control and wildtype LMP1-expressing cells were held in suspension for one hour, prior to plating onto either poly-L-lysine-coated plates, which supports cell adhesion via electrostatic interactions and thus does not engage integrins, or fibronectin-coated plates, which engages specific β1 integrins (α5β1, α3β1), and therefore activates downstream signalling. Protein was harvested after one, two, three and four hours, respectively, and subsequently analysed by immunoblotting with antibodies specific for the phosphorylated forms of ERK-MAPK and FAK, revealing the requirement for integrin-ligation in LMP1-mediated ERK-MAPK and FAK phosphorylation (Figure 7A).

Inside-out integrin signalling can serve to protect cells from suspension-induced apoptosis, also known as ‘anoikis’ [59]. Anoikis resistance can contribute to tumour progression by enabling the tumour cells to survive for longer periods of time whilst it breaks away from the primary tumour and migrates towards a secondary site. Anoikis has been shown to be mediated by β1 integrins [60,61], and since LMP1 can secrete fibronectin, the β1 integrin ligand, and in turn activate these integrins on the cell surface, it is possible that this may serve to protect LMP1-expressing epithelial cells from anoikis. In order to test this hypothesis, control and LMP1-expressing cells were recovered from monolayer culture using a weak trypsin:EDTA solution, and held in suspension for an hour by plating onto polyHEMA-coated dishes prior to assessing their viability by MTT assay. Figure 7B demonstrates that after only one hour in suspension, LMP1-expressing cells have an approximately 2.5-fold greater level of cell viability than their control counterparts. Addition of the β1 integrin blocking antibody, P5D2, reduced the viability of control cells only marginally (1.13-fold); however, in LMP1-expressing cells there was a nearly 2-fold reduction in cell viability, indicating that LMP1-mediated β1 integrin signalling goes some way to protecting epithelial cells from anoikis. Taken together, these findings support a role for LMP1-mediated β1 integrin signalling in the conversion to an EMT programme in order to promote survival and facilitate invasion and metastasis.

## 3. Discussion

NPC is a highly metastatic disease and one of the most invasive EBV-associated malignances [62]. Although the level of LMP1 expression is variable in NPC biopsies, it is widely believed to play a role in the early stages of disease pathogenesis [5]. LMP1 is able to transform B cells and rat fibroblasts in vitro [3] and causes morphological alterations in epithelial cells by activating a variety of cell signalling pathways commonly deregulated in cancer, including NF-κB1 and KF-κB2, ERK-MAPK, PI3K/Akt, JNK/SAP, p38-MAPK and activin A/TGFβ [6,7]. In vitro studies demonstrate the propensity of LMP1 to enhance epithelial cell adhesion and migration [7,31,37] and LMP1 has also been implicated in promoting EMT in a variety of different cell lines [8,30,32,33,34] and in NPC biopsies [9,35].

### 3.1. LMP1 Induces EMT in MDCK Cells via CTAR1 with Critical Roles for ERK-MAPK, PI3-Kinase, Src Family Kinases (SFKs) But Not TGFβ Signalling Pathways

EMT represents an important early step in the tumourigenic process, facilitating key steps in cancer development—invasion and metastasis [12]. During EMT, epithelial cells acquire a mesenchymal phenotype characterised by the loss of cell–cell adherens junctions, loss of cell polarity and actin cytoskeleton remodeling [15]. Simultaneously, cells undergoing EMT also increase the expression of mesenchymal markers and display enhanced migration, an ability that is mediated by alterations in cell–ECM interactions [18].

The morphological alterations observed in MDCK cells expressing wildtype and CTAR1+/2− LMP1, along with the appearance of actin stress fibres, support earlier observations of LMP1′s ability to induce an EMT phenotype in MDCK cells [8,9,10]. Furthermore, this phenomenon appears to be mediated via the CTAR1 signalling domain (Figure 1). A number of signalling pathways have been implicated in promoting EMT in vitro, including the pleiotropic integrin, TGFβ and MAPK signalling. There is significant crosstalk between these signalling pathways, which is mediated via transcription factors that are themselves induced by TGFβ-Smad signalling [18,19,20]. Interestingly, pharmacological inhibition of specific signalling pathways engaged by LMP1 revealed critical roles for ERK-MAPK, PI3K and Src family kinases (SFKs), but not TGFβ, despite its well-known role in EMT (Figure 2). However, albeit in a different cell system, previous studies have shown that LMP1 activates the non-Smad-dependent arm of activin A/TGFβ signalling [7]. Therefore, this inability of the canonical TGFβ inhibitor to reverse the morphological changes in LMP1-expressing MDCKs does not take into account the non-Smad arm of TGFβ signalling, which includes many of the same pathways that are constitutively activated by LMP1 as well—principally ERK-MAPK, PI3K/Akt, p38-MAPK and JNK/SAPK [63]. Indeed, in MDCK cells, TGFβ treatment can cooperate with oncogenic Ras, activating the Ras-Raf-MEK-ERK kinase module to induce an EMT [22]. Regardless, these findings may imply a role for integrin-mediated outside-in signalling in LMP1’s EMT programme, since integrin–ligand interactions are known to activate each of these downstream signalling pathways [64,65], although further studies are required to confirm this hypothesis.

### 3.2. Stable Expression of LMP1 in MDCK Cells is Accompanied by the Complete Loss of Epithelial Markers and Upregulation of Mesenchymal Markers

The transcriptional reprogramming that accompanies EMT results in downregulation of genes encoding cell–cell adhesion molecules, adherens junction proteins and associated signalling molecules, along with the concomitant upregulation of genes principally expressed in cells of mesenchymal origin. Cadherin switching is the classic characteristic of cancer-related EMT, whereby expression of epithelial cadherin (E-cadherin) is lost, but neuronal cadherin (N-cadherin) is switched on [19,20].

The observations presented here map this cadherin switching phenomenon to the CTAR1 domain of LMP1, along with the loss of multiple desmosomes, adherens and tight junctional proteins, and induction of numerous mesenchymal markers (Figure 3 and Figure 4; Supplementary Figures S3 and S4). A previous study showed that induction of the transcription factor, Twist, by LMP1 resulted in an EMT phenotype in MDCK cells, and also found a direct correlation between the expression of Twist and LMP1 in NPC tissues, with Twist expression correlating with metastatis [8]. Here, Twist induction by LMP1 is also mapped to the CTAR1 signalling domain (Figure 4B,C). Moreover, other transcription factors known to be involved in E-cadherin gene repression and important for EMT were also induced by LMP1 via the CTAR1 domain, including Slug, ZEB1 and SIP1/ZEB2 (Supplementary Figure S4). Interestingly, there was a very slight downregulation of Snail by LMP1, which may be an example of biological redundancy at play, or may be a reflection of the negative feedback loop that exists between Slug and Snail in order to maintain cellular homeostasis [66]. Nevertheless, the evidence for the profound transcriptional reprogramming that accompanies EMT in LMP1-expressing cells is compelling.

### 3.3. Microarray Analysis Identifies Deregulated Genes from Key Signalling Pathways Implicated in EMT, Including MAPK and Integrin Signalling Components

Detailed interrogation of the phenomenon by microarray analysis identified a number of EMT-related genes that are deregulated by LMP1 via the CTAR1 domain, including the transcriptional repressors already identified: Twist, Slug, Snail and ZEB1/2 (Figure 3 and Figure 4). Moreover, the microarray data highlighted groups of genes deregulated by CTAR1 that may be implicated in EMT, including those involved in MAPK and integrin signalling (Figure 3 and Table 1). In a study where MDCK cells expressing Snail, Slug or E47 transcription factors were genetically profiled, three genes were identified as being commonly upregulated by all three transcription factors: namely WNT5A, TIMP1 and SPARC [67]. Interestingly, each of these three genes are also upregulated by wildtype and CTAR1+/2− mutant LMP1-expressing cells (Supplementary Figure S1).

In the majority of cellular EMT models, the phenotypic conversion depends on non-cell-autonomous events and requires external signals. Factors acting at the tumour–stroma interface include growth factors and their receptors, including TGFβ, in addition to ECM-related molecules (collagens, fibronectin, integrins), as well as oncogenic pathways (Ras, Src), all of which appear to be critically involved in EMT. Therefore, it is conceivable that LMP1 is able to induce EMT through numerous signalling pathways converging at the cell surface with integrin-mediated outside-in signalling.

It has previously been shown that Raf1 induces the upregulation of Slug, which in turn downregulates occludin but could subsequently be prevented by treatment with the MEK1/2 inhibitor, UO126 [68]. Others have demonstrated a synergistic induction of E-cadherin ubiquitination via hyperactivation of TGFβ and MAPK signalling [69]. It is therefore possible that LMP1-mediated activation of the Ras-Raf-MEK-ERK kinase cascade may be involved in upregulating Slug expression, which in turn downregulates E-cadherin and occludin, leading to EMT progression. However, further studies are warranted to investigate the mechanisms underlying the LMP1-mediated EMT programme.

Expression of uPA and uPAR links ERK-MAPK signalling to the formation of focal adhesions and regulation of cell motility through differential expression of the integrin subunits. By binding uPA at the leading edge of the migrating cell, uPAR organises a cascade of extracellular proteases that facilitate cellular penetration of tissue boundaries [70]. Both wildtype and CTAR1+/2− LMP1-expressing cells predominantly induced the expression of genes encoding the α5 and αv integrin subunits (Supplementary Figures S2B and S5A). In human epidermal keratinocytes the α3, β1 and β4 integrin subunits have been identified as target genes downregulated upon activation of the E-cadherin transcriptional repressor, Slug [71]. Furthermore, of those integrins upregulated during wound healing, both α5 and αv possess E-box sequences, suggesting a possible regulatory mechanism involving Slug [71,72].

Activation of the PI3K/Akt pathway can lead to activation of the Rho GTPases and also cooperates with TGFβ signalling to affect EMT [45]. Akt phosphorylation and activation is known to inhibit the function of GSK3β, thereby inducing Snail expression [73]. Findings presented here demonstrate a role for the CTAR1 domain in LMP1-mediated Akt and GSK3β activity (Figure 5). Therefore, it would be interesting to further dissect this signalling pathway and any role it may play in LMP1-mediated EMT.

LMP1 is known to activate both canonical (NF-κB1/p105) and non-canonical (NF-κB2/p100) signalling pathways [47]. Findings presented here confirm the requirement of LMP1′s CTAR1 domain for activation of non-canonical NF-κB2 (Figure 5C,D).

In summary, these results demonstrate the role for the CTAR1 domain of LMP1 in activating numerous components of the MAPK and related signalling pathways that are implicated in EMT; however, further work is required to confirm whether these pathways are indeed involved in the LMP1-driven EMT progression.

### 3.4. Ligand-Induced β1 Integrin Signalling Facilitates LMP1-Mediated ERK and FAK Phosphorylation, and Protects Epithelial Cells from Anoikis

Further downstream analysis of these signalling pathways demonstrated the dependence on integrin–ligand interactions for LMP1-mediated ERK-MAPK and FAK signalling since p42/44 MAPK and FAK phosphorylation only occurred after plating onto the β1 integrin ligand, fibronectin (Figure 7A). β1 integrins can promote cell survival by transducing signals via FAK, Src, ERK-MAPK and PI3K/Akt [55,56,57,58]. This form of inside-out signalling can protect cells from anoikis, thereby contributing to tumour progression [59], and can be mediated by β1 integrins, which activates FAK at the sites of focal adhesion [60,61]. Moreover, LMP1 is able to activate β1 integrins on the surface of epithelial cells [7]. Findings presented here demonstrate that LMP1-expressing cells displayed greater viability when held in suspension for an hour when compared with their control counterparts, an effect that was ablated by abrogating β1 integrin signalling using a blocking antibody, P5D2 (Figure 7B), suggesting a role for LMP1-mediated signalling downstream of β1 integrins in protecting epithelial cells from anoikis, an effect that may help with facilitating invasion for the establishment of early secondary metastases.

### 3.5. EMT and NPC

The relevance of these studies to NPC is supported by previous work demonstrating an association between LMP1 and Snail expression in tumour biopsies and correlating the associated EMT with a more metastatic phenotype in vivo [9]. In a separate study, high levels of LMP1 expression were shown to be associated with poor clinical outcome [74]. More recent genetic analysis of NPC has revealed mutually exclusive somatic events in the ERBB-PI3K pathway and related these changes to more advanced clinical stage and shorter survival time [75]. This implicates the signalling pathways driving EMT in more aggressive disease and further supports the rationale for therapeutic targeting of these pathways.

Hypermethylation of the E-cadherin promoter has been shown to be induced in NPC tissues by LMP1 via DNA methyltransferase activation. The high incidence of EBV with the consistency of E-cadherin hypermethylation, particularly in undifferentiated and non-keratinising NPC, suggests a role for EBV in the hypermethylation [76]. However, at present it is unclear whether hypermethylation of the E-cadherin promoter is responsible for, or a consequence of, EMT. Therefore, comparing the methylation status of the E-cadherin promoter in pre- and post-EMT LMP1-expressing cells may address this question.

### 3.6. Current Working Hypothesis: LMP1-Mediated EMT Signals Converge at the Cell Surface with Integrin–Ligand Interactions

**Figure 8:**
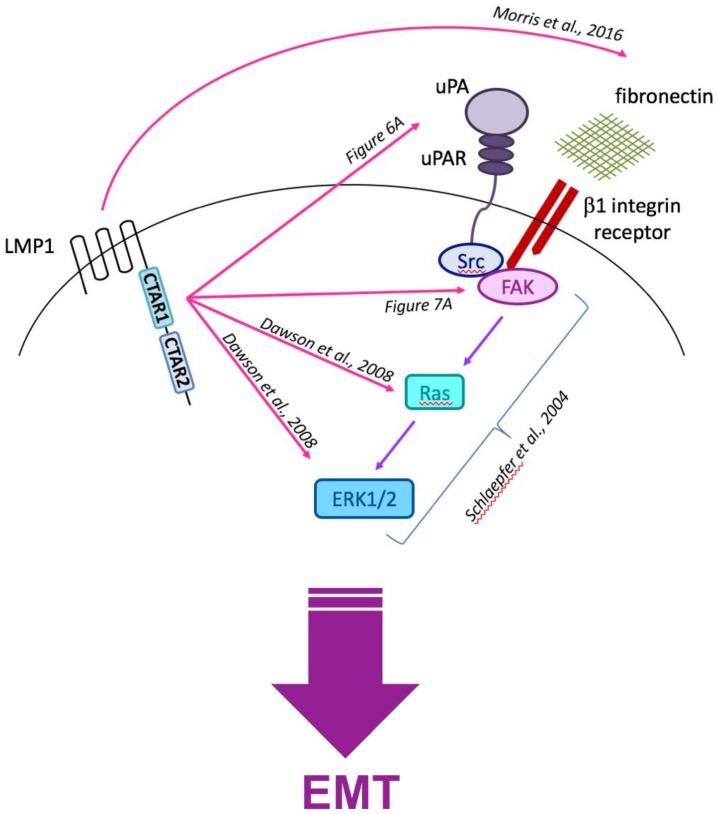
Schematic summarising the current working hypothesis for the LMP1-mediated EMT programme.

In addition to the previously published observations detailing LMP1’s ability to activate ERK-MAPK signalling [37] and induce the expression and secretion of fibronectin, thereby activating β1 integrins [7], the findings presented herein lend support to the ‘bigger picture’ idea that LMP1-mediated epithelial deregulation that results in EMT converges at the cell surface with the coordinated ligand-induced activation of β1 integrins, the concomitant recruitment of uPAR, resulting in the phosphorylation and activation of the Src-FAK complex and subsequent downstream activation of the Ras-Raf-MEK-ERK kinase module, propagating a pro-survival, pro-metastatic signal for the long-term survival and propagation of the tumour. Figure 8 summarises the current working hypothesis.

In summary, we have confirmed and further characterised the role of LMP1 in EMT, demonstrating the key contribution of the CTAR1 domain and the role of ERK-MAPK, PI3K and Src family kinase signalling pathways. As we begin to better understand the contribution of EBV and LMP1 to the development of NPC [77] and define the role of EBV strain variation [78], this study highlights the importance of well-defined cell model systems to reveal underlying mechanisms. Given the key role of EMT in the metastatic process and our lack of understanding of how EBV contributes to tumour spread in NPC, this study provides important insights that could guide the use of various therapeutic interventions.

## 4. Materials and Methods

### 4.1. Cell Lines

The canine kidney epithelial cell line, MDCK, has been described previously [79]. MDCK cells expressing the neomycin resistance gene (*neoR*), wildtype LMP1, CTAR1+/2−, CTAR1−/2+ and the CTAR1−/2− double-mutant derivatives have been described previously [37]. All cell lines were cultured in high glucose DMEM (Sigma, Welwyn Garden City, UK) supplemented with 5% fetal calf serum (FCS), 1% L-glutamine, and G418 (Invitrogen, Rugby, UK; 400 μg/mL) to select for cells containing the neomycin resistance cassette.

### 4.2. Treatment with Selective Pharmacological Inhibitors

Cells were seeded at 1 × 10^5^ cells/well in 6-well plates and allowed to adhere overnight in complete growth medium. Prior to treatment, cells were washed twice with media containing 0.5% FCS to remove any residual serum and cytokines present, then incubated in medium containing 0.5% FCS containing the appropriate concentration of specific pharmacological inhibitors, as outlined in Supplementary Table S4, or using the carrier solvent DMSO as a control, and incubated for 24 h prior to taking phase contrast images on a Zeiss digital microscope.

### 4.3. Transfections

For transient report activity, 2 × 10^5^ MDCK cells were transfected with 1 μg of the relative luciferase reporter construct, or negative control plasmid and 0.25 μg Renilla plasmid using Lipofectamine and Plus Reagent (Invitrogen). A complete list of plasmids is outlined in Supplementary Table S5. Cells were harvested in passive lysis buffer (Promega, Southampton, UK) 24 h later and luciferase activity assayed on a Victor plate reader. Data is presented as a histogram displaying the relative fold changes in the levels of promoter reporter activity. Statistical significance was determined using the student’s *t*-test after first determining whether the sample variance was equal or unequal by performing an F-test.

### 4.4. Immunofluorescence Microscopy

Cells grown on Teflon-coated microscope slides (Hendley-Essex, Loughton, UK) were processed for imaging as described previously [37]. Samples were incubated with primary antibodies followed by secondary antibodies in 20% heat-inactivated goat serum (HINGS). A complete list of antibodies is outlined in Supplementary Table S6. Slides were mounted with coverslips using DABCO anti-fade medium containing DAPI (Invitrogen). Images were obtained using the 40x oil objective on a Leica inverted fluorescence microscope and processed using OpenLAB (ver.X.0) software (Agilent, Cheshire, UK).

### 4.5. Western Blotting

Cells were lysed in 500 μL RIPA buffer (50 mM Tris (pH 7.4), 150 mM NaCl, 1 mM EDTA, 1% (*v*/*v*) NP-40, 0.25% (*w*/*v*) sodium deoxycholate, dissolved in sterile distilled water) and briefly sonicated. 30 μg of total protein lysate was separated by SDS-PAGE and transferred to nitrocellulose. Membranes were blocked in 5% non-fat powdered milk in PBS, then incubated with primary antibodies (Supplementary Table S7). After washing, blots were probed with goat anti-mouse peroxidase (1:2000) or goat anti-rabbit peroxidase (1:2000; Dako, Cambridge, UK), and then developed using chemiluminescence reagents (ECL; Perkin Elmer, Beaconsfield, UK). Membranes were stripped in 0.1 M glycine pH 2.9 for 30 min, washed in PBS-Tween, blocked and re-probed with the next antibody as described above.

### 4.6. Reverse-Transcriptase Polymerase Chain Reaction (RT-PCR)

RNA was extracted from subconfluent cell cultures using Trizol Reagent (Invitrogen, UK) and RNA concentration for each sample was quantified on a Nanodrop ND-1000 spectrophotometer (Labtech International, Heathfield, UK). cDNA was synthesised using either Oligo dT (Invitrogen, UK) or random primers (Promega), and amplified by RT-PCR with GoTaq Green polymerase according to the manufacturer’s instructions. For loading control, a 452 bp GAPDH housekeeping gene was amplified. A full list of 5′ and 3′ primer combinations with their respective annealing temperatures are outlined in Supplementary Table S8. Human-specific primer combinations were used where canine-specific primer combinations could not be designed or were used in previously published papers.

### 4.7. Non-Radioactive Kinase Assays

p44/42 MAP kinase and Akt kinase activity was assayed using commercially available kits according to the manufacturer’s instructions (Cell Signalling Technology, Danvers, MA, USA). Briefly, total cell lysates were collected from serum-starved subconfluent cultures prior to overnight incubation at 4 °C with immobilised antibodies to Thr202/Tyr204-phosphorylated p44/42 MAPK and Akt, respectively. Washed immune complexes were suspended in kinase buffer supplemented with 200 μM adenosine-5′ tri-phosphate (ATP) and 2 μg of Elk-1 fusion protein or GSK-3 fusion protein, respectively. The reactions were heat-activated prior to termination with 25 μL 3× gel sample buffer [187.5 mM Tris-HCL (pH 6.8 at 25 °C), 6% (*w*/*v*) SDS, 30% glycerol, 150 mM dithiothreitol (DTT), 0.03% (*w*/*v*) bromophenol blue]. Subsequently, samples were separated by SDS-PAGE and immunoblotted using antibodies to Ser383-phosphorylated Elk and Ser21/9-phosphorylated GSK-3α/β, respectively, prior to detection with anti-rabbit HRP-conjugated secondary antibody.

### 4.8. MTT Assay

A 12-well plate was coated with 20 μg/mL poly (2-hydroxyethyl methacrylate) diluted in ethanol (polyHEMA; Sigma) and air-dried overnight in a laminar airflow cabinet. Cells were seeded at 1 × 10^4^ cells/well, then incubated at 5% CO_2_, 37 °C for one hour. Cell suspensions were aspirated in triplicate universal tubes and centrifuged (1500 rpm, 5 min) and the pellet resuspended in 100 μL PBS with 10% thiazolyl blue tetrazolium bromide (MTT; Sigma). The cell suspension:MTT mixture was incubated for two hours in the dark, after which point the reaction was stopped by addition of 100 μL DMSO, and then placed on a shaker in the dark for 15 min. Absorbance was read at 560 nm on a plate reader (Promega).

### 4.9. Gene Expression Profiling

Microarray samples were prepared in biological triplicate and RNA extraction was carried out using Trizol Reagent as before, prior to purification using the RNeasy Mini Kit according to the manufacturer’s instructions (Qiagen, Manchester, UK). After quantification on a Nanodrop ND-1000 and quality check by agarose gel electrophoresis, samples were loaded onto Affymetrix GeneChip^®^ Canine Genome 2.0 array chips (Affymetrix, High Wycombe, UK). Probe level quantile normalisation [80] and RMA (robust multi-array analysis) [81] were performed using the affy package of the Bioconductor project (http://www.bioconductor.org). Probe sets differentially expressed between two groups were identified using the rank products method [43,44] with the criteria of percentage of false-positives less than 10% and absolute fold change ≥1.5. Top 1021 probe sets differentially expressed among the five groups were identified by multiclass SAM analysis [42] with the criteria of fold change greater than 2% and 0% FDR (false discovery rate). Gene expression heatmaps were generated using dChip (http://www.dchip.org) with the default settings. The raw and normalized microarray data are available from GEO (GSE111340).

## 5. Conclusions

The observations presented here demonstrate the ability of LMP1 to induce an EMT phenotype in MDCK cells via its CTAR1 signalling domain. Use of selective pharmacological inhibitors showed critical roles for ERK-MAPK, SFK and PI3-Kinase, but not TGFβ, in LMP1-mediated EMT. Microarray analysis identified groups of EMT-related genes commonly deregulated by wildtype and CTAR1+/2− mutant LMP1, as well as many genes involved in MAPK and integrin signalling. LMP1-mediated ERK-MAPK and FAK phosphorylation was shown to require ligation of β1 integrins with its cognate ligand, fibronectin. Finally, LMP1 was able to protect epithelial cells held in suspension from anoikis—an effect that was lost upon inhibition of β1 integrin signalling using a blocking antibody. Overall, this study provides compelling evidence for the role of the CTAR1 signalling domain of LMP1 in promoting an EMT in MDCK cells and an intriguing role for signals converging at the cell surface in facilitating invasion and metastasis. Future work will involve further delineation of these signalling phenomenon in LMP1-expressing cells, which may be important in the early stages of NPC pathogenesis and could therefore prove useful as putative therapeutic targets for treatment of NPC.

## Figures and Tables

**Table 1:** Table summarizing the key gene changes mapped to the CTAR1 domain of LMP1 relating to the EMT phenotype, mitogen-activated protein kinase (MAPK) and integrin signalling.

Category	Gene	Fold-Change in CTAR+/2− LMP1
EMT phenotype	*E-cadherin*	−50.5
	*N-cadherin*	7.3
	*Desmocollin 2*	−7.2
	*Desmoglein 3*	−33.0
	*Plakoglobin*	−5.3
	*Plakophilin 3*	−7.4
	*Occludin*	−26.4
	*Periplakin*	−30.2
	*Claudin 3*	−10.0
	*ZEB1*	199.3
	*ZEB2*	12.0
	*Snail homologue 2*	2.7
	*Podoplanin*	9.8
MAPK signalling	*MAPK1/3*	2.2
	*RASA1*	4.0
	*RRAS2*	3.3
	*MAP3K4*	2.4
	*IL-1α*	2.2
	*AKT3*	3.8
Integrin	*α5*	9.1
	*α6*	−3.2
	*β1*	2.4
	*β4*	−3.8
	*Fibronectin*	22.8

## Supplementary Materials

The following are available online at http://www.mdpi.com/2072-6694/10/5/130/s1, Figure S1: Heatmaps generated to examine the impact of LMP1 and LMP1-CTAR inactivating mutant expression in MDCK cells; Figure S2: Heatmaps generated to examine the impact of LMP1 and LMP1-CTAR inactivating mutants on the expression of genes encoding adherens junction and focal adhesion proteins; Figure S3: Intracellular expression and distribution of cell adhesion proteins in MDCKs expressing wildtype and CTAR-inactivating LMP1 mutants; Figure S4: Validation of EMT markers in MDCKs expressing wildtype and CTAR-inactivating LMP1 mutants; Figure S5: Characterisation of integrin expression in MDCKs expressing wildtype and CTAR-inactivating LMP1 mutants; Table S1: Genes (with Entrez ID) upregulated in array compared with control cells; Table S2: Genes (with Entrez ID) downregulated in array compared with control cells; Table S3: Adherens junction and focal adhesion signalling genes differentially regulated by LMP1; Table S4: List of pharmacological inhibitors used; Table S5: Complete list of plasmids used; Table S6: Complete list of antibodies used in immunofluorescence; Table S7: Complete list of antibodies used in Western blotting; Table S8: Complete list of 5′ and 3′ primer combinations used.
