# Supplementary figures and images for: The EBV-Encoded Oncoprotein, LMP1, Induces an Epithelial-to-Mesenchymal Transition (EMT) via Its CTAR1 Domain through Integrin-Mediated ERK-MAPK Signalling
Source: Cancers (Basel). 2018 May 1;10(5):130. doi: 10.3390/cancers10050130 (PMC5977103; doi:10.3390/cancers10050130)

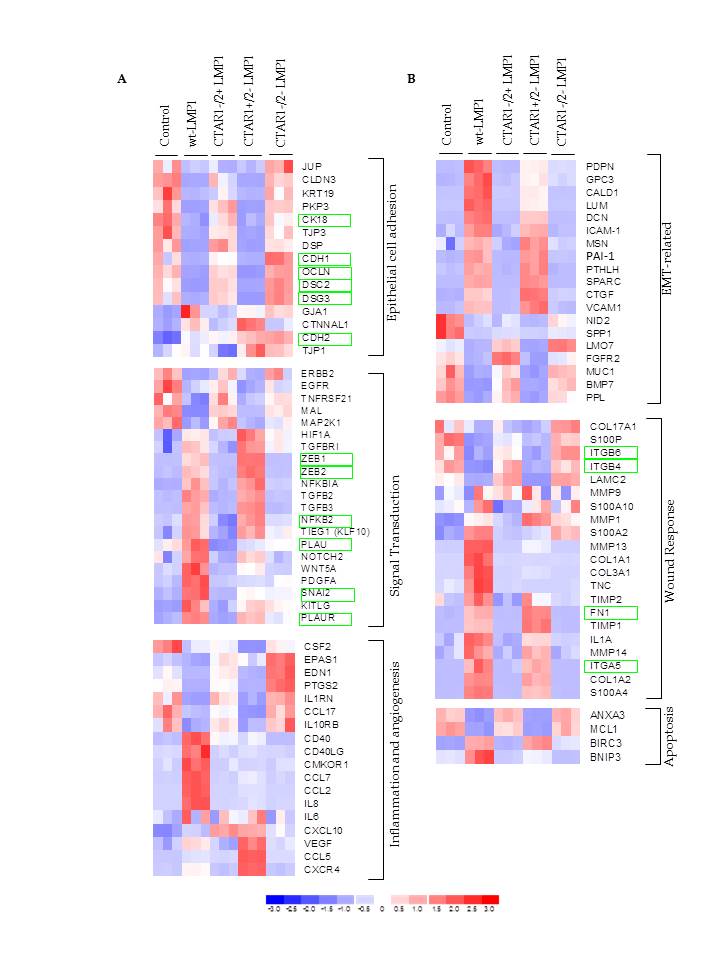

Supplement: Supplementary file 1 [file cancers-10-00130-s001.zip › Morris, Laverick et al., 2018 - Supplementary Figure S1.JPG]

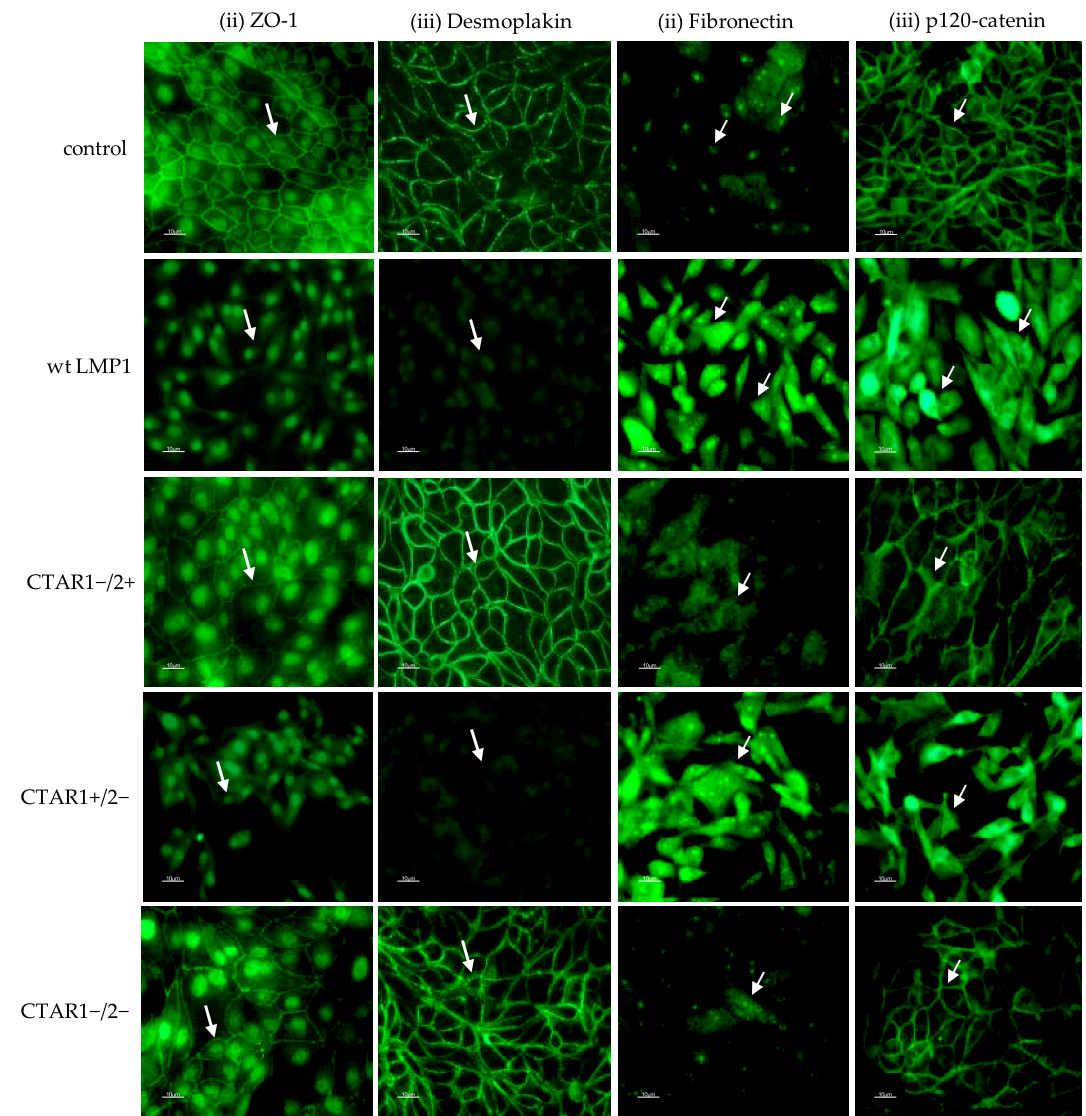

Supplement: Supplementary file 1 [file cancers-10-00130-s001.zip › Morris, Laverick et al., 2018 - Supplementary Figure S3.png]

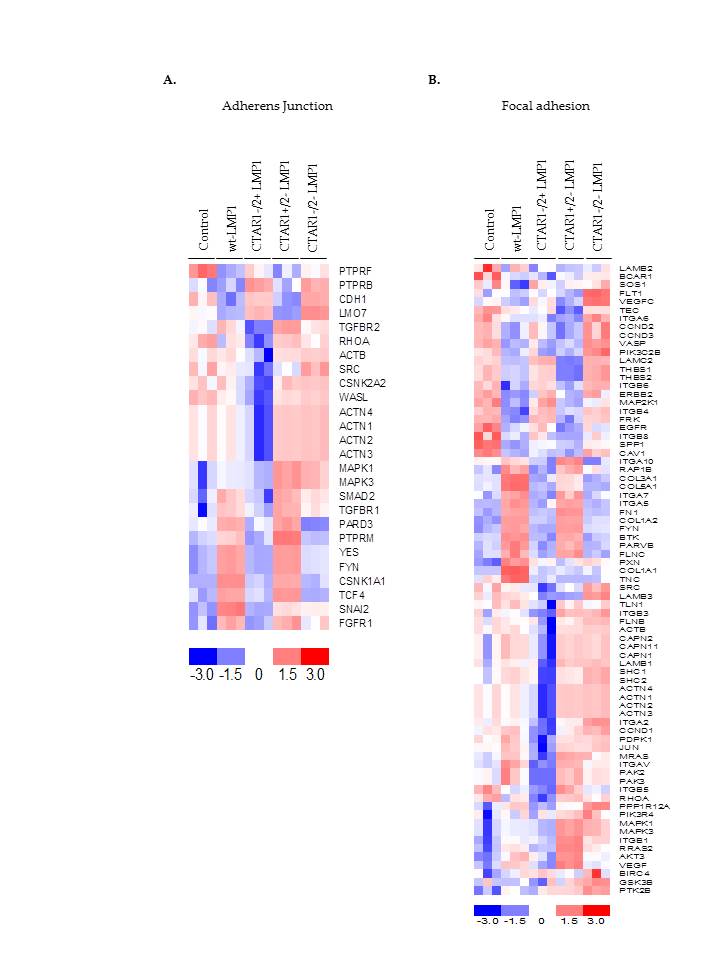

Supplement: Supplementary file 1 [file cancers-10-00130-s001.zip › Morris, Laverick et al., 2018 - Supplementary Figure S2.JPG]

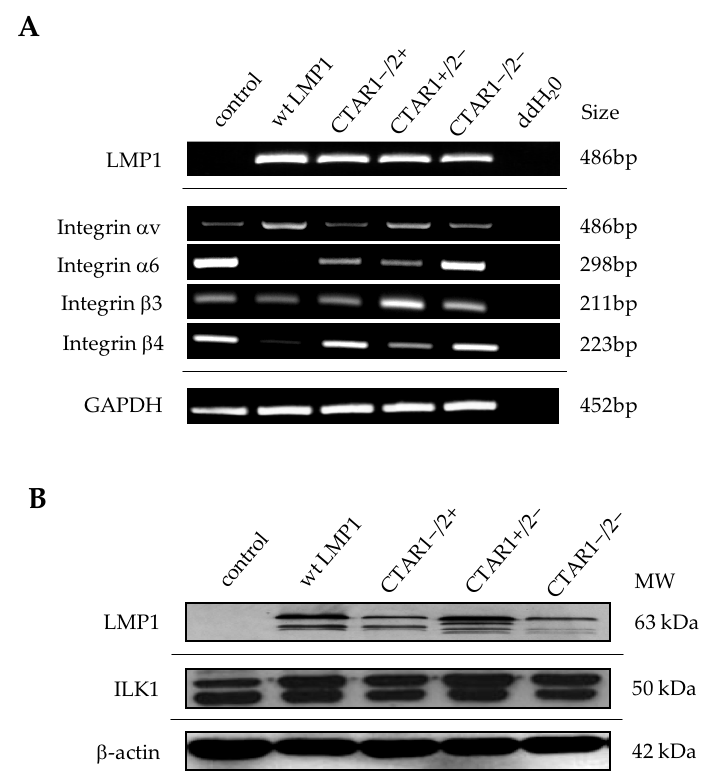

Supplement: Supplementary file 1 [file cancers-10-00130-s001.zip › Morris, Laverick et al., 2018 - Supplementary Figure S5.png]

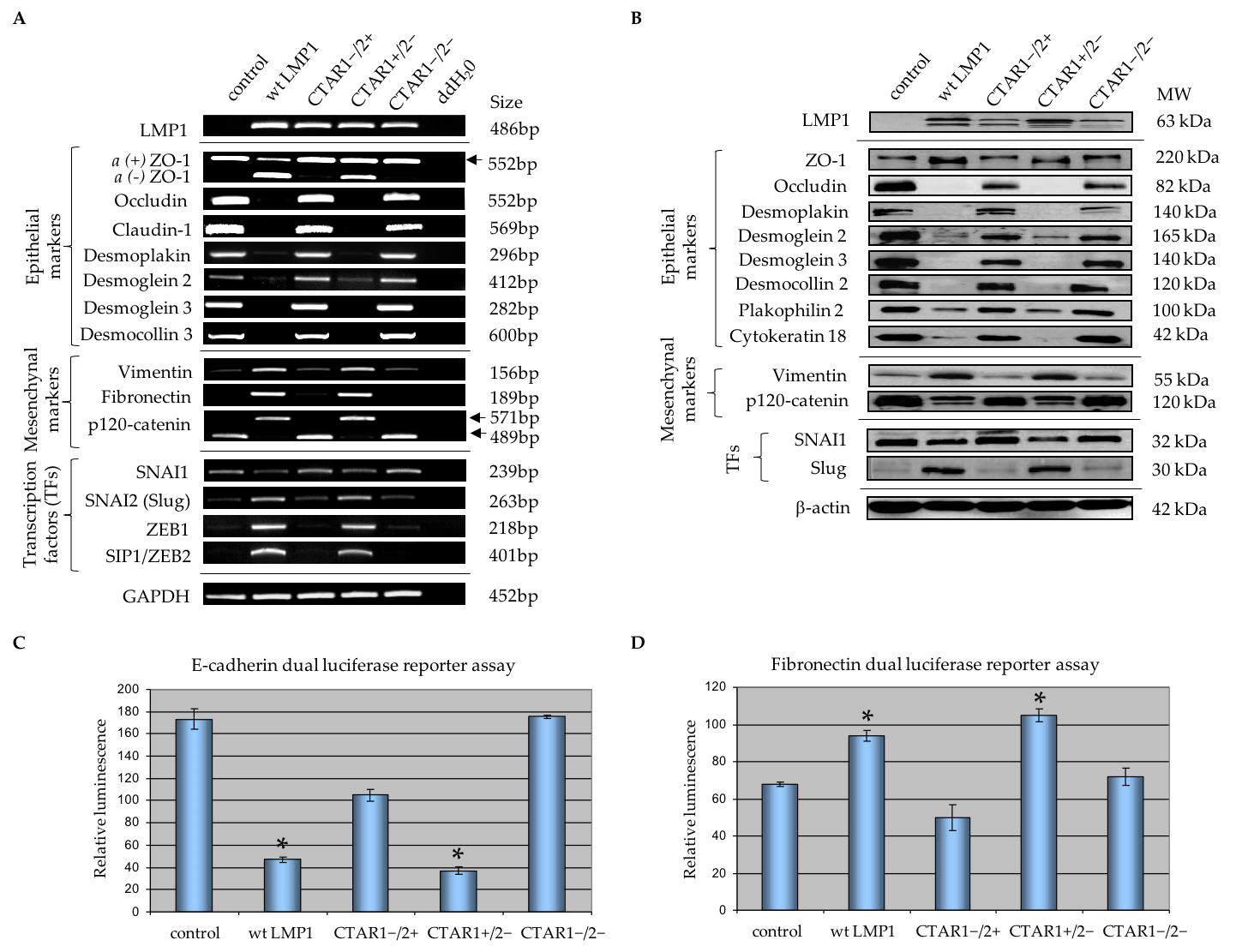

Supplement: Supplementary file 1 [file cancers-10-00130-s001.zip › Morris, Laverick et al., 2018 - Supplementary Figure S4.png]
